# Isolation of human bone marrow stromal cells from bone marrow biopsies for single-cell RNA sequencing

**DOI:** 10.1016/j.xpro.2021.100538

**Published:** 2021-05-12

**Authors:** Hélène F.E. Gleitz, Inge A.M. Snoeren, Stijn N.R. Fuchs, Nils B. Leimkühler, Rebekka K. Schneider

**Affiliations:** 1 Department of Hematology, Erasmus MC, Rotterdam, 3015GD, the Netherlands; 2 Current address: Department of Developmental Biology, Erasmus MC, Rotterdam, 3015GD, the Netherlands; 3 Oncode Institute, Erasmus MC, Rotterdam, 3015GD, the Netherlands; 4 Department of Hematology, Oncology, Hemostaseology, and Stem Cell Transplantation, Faculty of Medicine, RWTH Aachen University, Aachen 52074, Germany; 5 Institute for Biomedical Engineering, Department of Cell Biology, Rheinisch-Westfälische Technische Hochschule Aachen University, Aachen, Germany

**Keywords:** Cell Biology, Cell isolation, Flow Cytometry/Mass Cytometry, Cancer

## Abstract

Bone marrow (BM) mesenchymal stromal cells play an important role in regulating stem cell quiescence and homeostasis; they are also key contributors to various hematological malignancies. However, human bone marrow stromal cells are difficult to isolate and prone to damage during isolation. This protocol describes a combination of mechanical and enzymatic isolation of BM stromal cells from human BM biopsies, followed by FACS sorting to separate stromal sub-populations including mesenchymal stromal cells, fibroblasts, and Schwann cells for single-cell RNA sequencing.

For complete details on the use and execution of this protocol, please refer to Leimkühler et al. (2020).

## Highlights

• Combination of enzymatic and physical homogenization methods for tissue dissociation
• Isolation of viable bone marrow stromal cells from human trephine bone marrow biopsies
• Preparation of FACS-sorted human stromal cells for single-cell RNA sequencing experiments

## Graphical abstract

**Figure:**
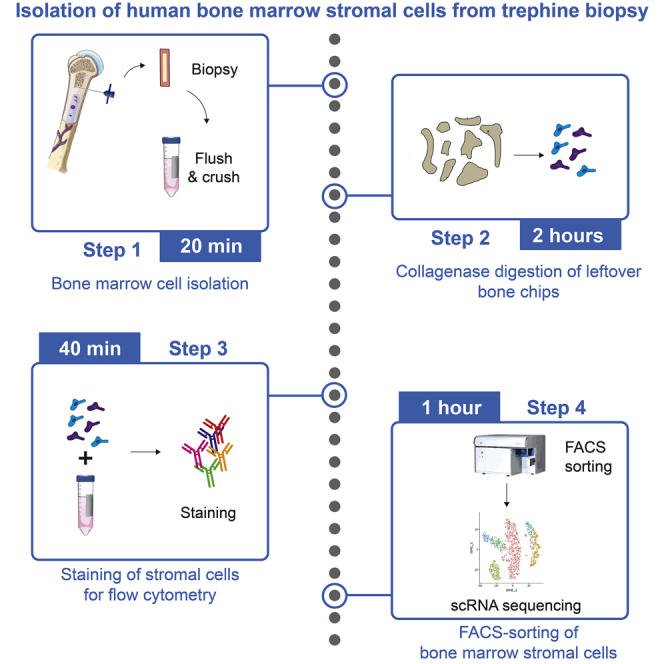

## Before you begin

### Background

**Figure 1:**
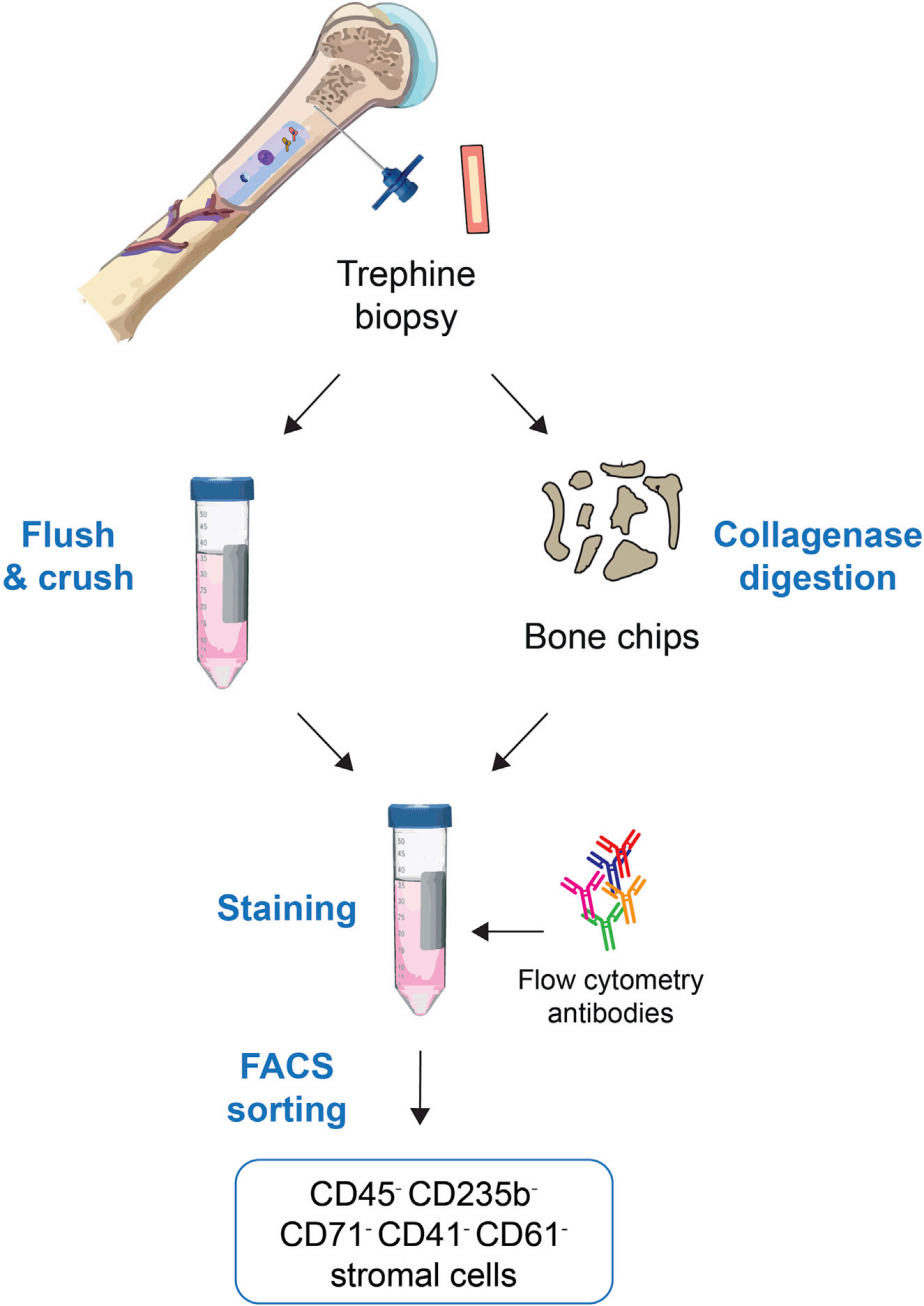
Overview schematic detailing the main protocol steps to isolate human BM stromal cells A small piece of trephine biopsy is flushed, crushed, and enzymatically digested with Collagenase II to release stromal cells, which are stained for flow cytometry and subsequently FACS-sorted. Hem. cells: hematopoietic cells, FACS: Fluorescence-activated cell sorting.

This protocol describes the isolation of bone marrow (BM) stromal cell populations from fresh BM biopsies for single-cell RNA sequencing analysis. The procedure is divided into 4 main and sequential steps (Figure 1): 1) the biopsy is flushed and crushed, 2) bone chips are digested with collagenase, 3) cells are stained for fluorescence-activated cell sorting (FACS), and 4) FACS of bone marrow stromal cells. This protocol was specifically developed for biopsies taken from primary myelofibrosis patients but could be adapted for samples from different origins.

Unprocessed BM biopsies are obtained approximately 1–2 h after successful BM biopsy. BM biopsies should be obtained and processed as soon as possible to maximize cell number and viability. Although previous techniques for the isolation and use of BM stromal cells *in vitro* exist, this protocol describes the isolation of human BM stromal cells from trephine biopsies specifically for downstream single-cell RNA sequencing analysis. In this protocol, BM stromal cells are negatively selected from a large, heterogeneous mix of crushed and flushed BM cells by staining "contaminating” cells with hematopoietic, megakaryocytic, and erythroid markers. The use of FACS enables the identification of small cell populations reproducibly.

### Cell buffers and antibody master mix preparation

**Timing: 20 min**

1. See Materials & Equipment for preparation of the Cell Isolation buffer, Collagenase II solution, antibody mastermix and sorting buffer. Ensure that all solutions are kept on ice or at 4°C until use.
2. Cool a centrifuge set up for 50 mL Falcon tubes, such as the Thermo Scientific™ Sorvall™ Legend™ XT, down to 4°C.
3. Warm a shaking incubator to 37°C.

***Alternatives:*** a water bath set to 37°C can also be used, provided there is gentle agitation of the sample.

## Key resources table

**Table:** 

REAGENT or RESOURCE	SOURCE	IDENTIFIER
**Antibodies**		

FITC anti-human CD45 Antibody	BioLegend	Cat#368508; RRID: AB_2566368
FITC anti-human CD235ab Antibody	BioLegend	Cat#306610; RRID: AB_756046
FITC anti-human CD71 Antibody	BioLegend	Cat#334104; RRID: AB_2201482
FITC anti-human CD41 Antibody	BioLegend	Cat#303704; RRID: AB_314374
FITC anti-human CD61 Antibody	BioLegend	Cat#336404; RRID: AB_1227580
7-AAD Viability Staining Solution	BioLegend	Cat#420404

**Biological samples**		

Bone marrow trephine biopsy	Department of Pathology, Erasmus MC, The Netherlands	N/A

**Chemicals, peptides, and recombinant proteins**		

Collagenase II	Invitrogen	Cat#17101015
Fetal bovine serum (FBS)	Sigma-Aldrich	Cat#F7524
Phosphate buffered saline (PBS)	Gibco	Cat#10010-023
Dulbecco’s Modified Eagle Medium (DMEM), high glucose	Gibco	Cat#41965039
Alpha Modified Eagle Medium (αMEM)	STEMCELL Technologies	Cat#36450
Penicillin-streptomycin (P/S)	Gibco	Cat#15140122

**Software and algorithms**		

BD FACS Diva Software	BD Biosciences	https://www.bdbiosciences.com
FlowJo v.10	Tree Star, LLC	RRID:SCR_008520

**Other**		

Porcelain mortar and pestle	Fisherbrand	Cat#FB961C
70 μm Cell strainers	Corning	Cat#CLS431751-50EA
Parafilm	Sigma-Aldrich	Cat#P7793-1EA
Countess II FL Automated Cell Counter	Thermo Fisher Scientific	Cat#AMQAF1000
5 mL Falcon® Round-Bottom Tube with Cell Strainer Cap	Falcon	Cat#352235
Thermo Scientific™ Sorvall™ Legend™ XT	Thermo Fisher Scientific	Cat#75004538
BD FACSAria II	BD Biosciences	N/A

## Materials and equipment

**Table:** Cell Isolation buffer (PBS/2% FBS)

Reagent	Volume	Final concentration
Phosphate buffered saline (PBS), sterile, pH 7.4	490mL	-
Fetal bovine serum (FBS), heat-inactivated and sterile-filtered	10mL	2%
**Total**	500mL	-

Storage: Keep at 4°C until use. The cell isolation buffer can be stored for up to 7 days at 4°C, but we recommend making fresh buffer for every new sample.

**Table:** Collagenase II solution

Reagent	Volume	Final concentration
alphaMEM, sterile, 10% FCS, 1% P/S	10 mL	-
Collagenase II	10mg	1mg/mL
**Total**	10mL/biopsy	-

Prepare fresh and use immediately.

**Table:** Antibody master mix for FACS-sorting

Reagent	Volume of antibody per sample	Dilution
FITC-CD45	1μL	1:100
FITC-CD235a	1μL	1:100
FITC-CD71	1μL	1:100
FITC-CD41	1μL	1:100
FITC-CD61	1μL	1:100
**Total**	5μL/sample	-

Storage: Keep at 4°C in the dark until use. This master mix can be stored at 4°C for no longer than 24 h.

**Table:** Sorting buffer

Reagent	Volume	Final concentration
DMEM, high glucose (90%)	9mL	-
Fetal bovine serum (FBS), heat-inactivated and sterile-filtered	1mL	10%
**Total**	10mL	-

Storage: Keep at 4°C until use. The sorting buffer can be stored for up to 7 days at 4°C, but we recommend making fresh buffer for every new sample.

**CRITICAL:** Ensure that all prepared buffers are kept at 4°C until use.

**CRITICAL:** Ensure that the antibody master mix is kept at 4°C in the dark until use.

***Alternatives:***

**Sorting buffer:** This can also be substituted with PBS + 10% FBS.

## Step-by-step method details

### Isolation of BM cells and stromal cells from trephine biopsy

**Timing: ∼20 min per biopsy**

This step describes the isolation of hematopoietic and non-hematopoietic BM cells by flushing and crushing the trephine biopsy. This sample includes both a bone fragment and bone marrow cells and is usually taken to identify hematological abnormalities. The trephine biopsy includes a bone fragment that needs to be flushed/crushed in order to isolate BM cells. This step should be performed as quickly as possible after the biopsy has been taken to maximize cell viability and quality for downstream analysis and applications. Additionally, it is recommended to work quickly and efficiently, and to keep cell fractions at 4°C if possible.

**Figure 2:**
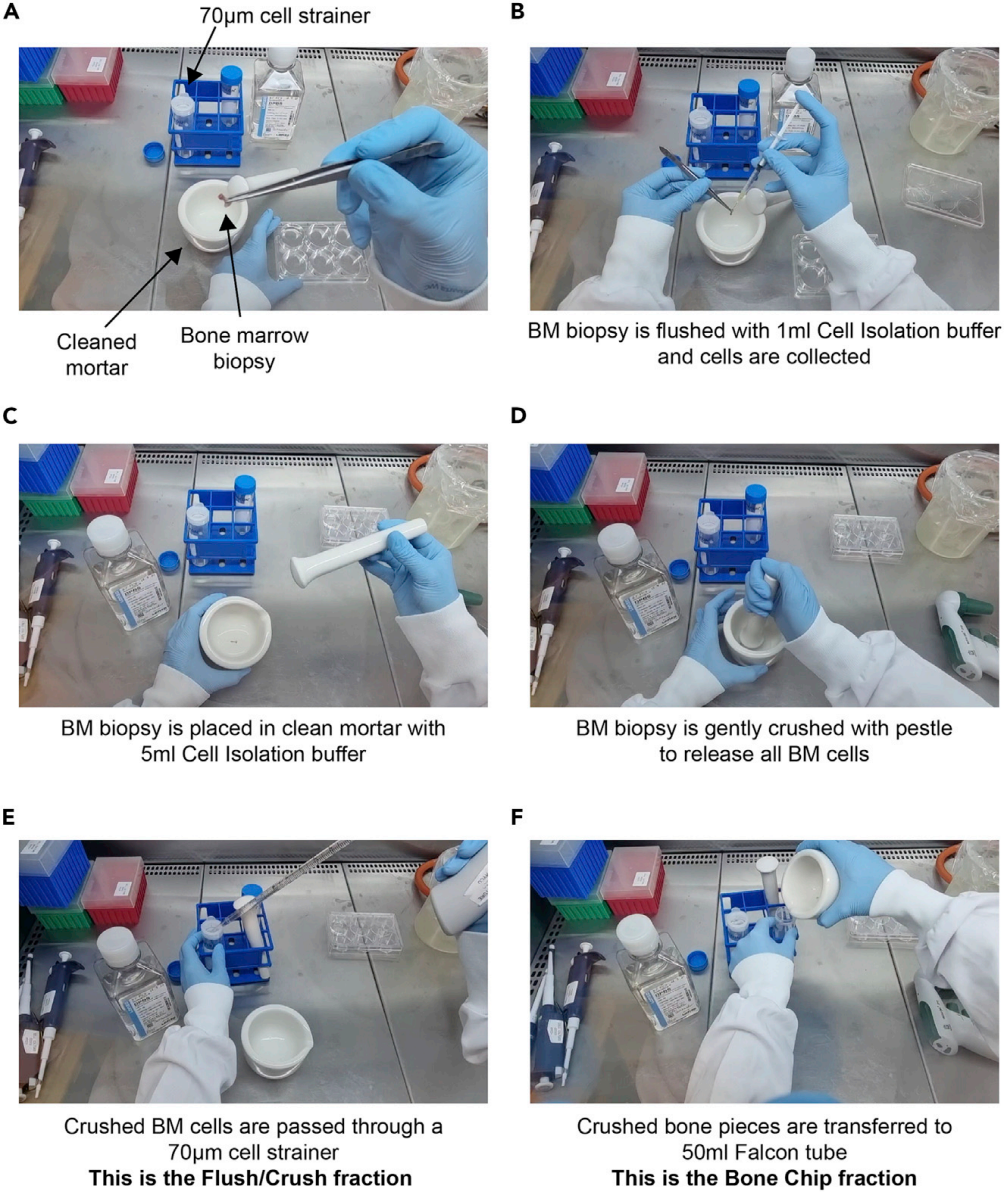
Preparation of the flush/crush and bone chips fractions from the bone marrow biopsy (A) A cleaned mortar/pestle and a 70 μm cell strainer is placed on a 50 mL Falcon tube. (B) The BM biopsy is flushed with a 1 mL insulin syringe or using a P1000 pipette tip into the cleaned mortar (or directly through the strainer). (C) BM biopsy is placed in clean mortar with 5 mL Cell Isolation buffer (2%FBS/PBS). (D) BM biopsy is gently crushed using the pestle, gently tapping directly onto the bone fragments. (E) Crushed cells are transferred to the 70 μm cell strainer and collected with the Flushed fraction. (F) Remaining crushed bone fragments are transferred to a new 50 mL falcon tube and will be digested with Collagenase II enzyme.
3. Place a 70 μm cell strainer over a 50 mL Falcon tube to collect flushed/crushed BM cells (Figure 2A).
4. Using a P1000 pipette or a 1 mL Insulin syringe, carefully flush the BM biopsy with 1 mL Cell Isolation buffer (PBS/2% FBS) through the cell strainer into the 50 mL Falcon tube [**This will be referred to as the Flush/Crush Fraction**] (Figure 2B). Repeat this step twice.
5. Place the remaining BM biopsy into a sterile mortar and add 5 mL Cell Isolation buffer (Figure 2C).
6. Crush the BM biopsy to release the BM cells by gently grinding the pestle onto the biopsy fragment (Figure 2D).

***Note:*** We recommend gently tapping the pestle onto the bone fragments to release as many cells as possible into the buffer.

***Note:*** To maximise the number of cells that can be analysed downstream, avoid brisk movements that could result in spills and loss of cells.

1. Clean a biohazard safety cabinet or sterile working area, as well as any small equipment (pipettes, tweezers) with 70% ethanol.
2. Clean a mortar and pestle with 70% ethanol. Remove the ethanol from the mortar and pestle and allow it to air dry (∼5 min) in a sterile biohazard safety cabinet. Rinse the mortar and pestle with sterile PBS just prior to use (Figure 2A).

7. Carefully transfer the buffer (approximately 5 mL) from the mortar through the cell strainer into the 50 mL Falcon tube used in step 4 [**Flush/Crush Fraction**] (Figure 2E).
8. Centrifuge the **Flush/Crush Fraction** tube at 300 **×** *g* for 5 min at 4°C and discard supernatant.
9. Resuspend the cell pellet in 300 μl Cell Isolation buffer. It is best to resuspend the pellet gently, in order to prevent clumping. This tube [**Flush/crush Fraction**] is kept on ice until further processing.

***Note:*** If clumps do occur during resuspension, filter the cell suspension through a new 70μm cell strainer. However, this leads to a loss of cells, so it should be avoided if possible.

10. Transfer the remaining bone fragments (bone chips) from the mortar into a second sterile 50 mL Falcon tube [**Bone Chip Fraction**] (Figure 2F). Bone chips will be enzymatically digested with Collagenase II solution, which is used to disrupt extracellular matrix and collagen fibers, in order to release additional stromal cells from the bone fragments.
11. Rinse the mortar and the cell strainer used in steps 4 and 7 with the prepared 10 mL Collagenase II solution and combine with the **Bone Chip Fraction** tube. Proceed directly to step 12.

### Isolation of BM stromal cells from bone chips using collagenase digestion

**Timing: ∼2 h**

This step is performed to release BM stromal cells that remain in the bone fragments that have previously been crushed using enzymatic digestion. This will increase the number of stromal cell populations that are detected in single-cell RNA sequencing and provide the researcher with a broader dataset to analyze.

12. Seal the **Bone Chip Fraction** 50 mL Tube containing bone chips and Collagenase II solution with Parafilm to prevent spills or contamination during incubation step 13.
13. Place the tube in a pre-warmed, shaking incubator and incubate the **Bone Chip Fraction** tube for 90 min at 37°C under gentle agitation.
14. After digestion, let the bone chips settle to the bottom of the 50 mL falcon and carefully transfer supernatant through a 70 μm cell strainer into a new 50 mL Falcon tube.
15. Wash the digested bone chips with 10 mL Cell Isolation buffer and combine supernatant with the new tube used in step 14. Repeat this step and combine supernatant.

***Note:*** This step significantly reduces cell loss and ensures the transfer of the greatest number of cells for downstream analysis.

16. Centrifuge cells at 300 **×** *g* for 5 min at 4°C and discard supernatant.
17. At this point, the cell suspension obtained from bone chips can be combined in a 50 mL Falcon tube with the cell suspension obtained from the flushed/crushed fraction in step 9.

***Optional:*** Cell counts and viability checks can be performed after step 17 using Trypan Blue and a Countess II FL Automated Cell Counter, or alternative cell counter. Cell counts are useful to determine cell viability (at least >60% live cells) and the total numbers of cells isolated from a specific biopsy and can be used to estimate sorting duration and general cell fitness.

**CRITICAL:** Except for the collagenase II digestion, always use solutions stored at 4°C to maintain cell viability.

### Staining of BM stromal fraction for FACS sorting

**Timing: 40 min**

This step is performed to stain the human BM cell suspension with flow cytometry-specific antibodies to isolate human BM stromal cells using fluorescence-activated cell sorting (FACS). To obtain a more specific BM stromal cell population, we use CD41 and CD61 antibodies to exclude megakaryocytes, CD71 and CD235a to exclude erythroid cells and CD45 to exclude the majority of hematopoietic cells.

18. Centrifuge the combined cell suspensions in a 50 mL Falcon tube [**Flushed/Crushed Fraction** and **Bone Chip Fraction**] at 300 **×** *g* for 5 min at 4°C.
19. Discard supernatant and gently resuspend the cell pellet in 110 μL Cell Isolation buffer.
20. Aliquot 10 μL cell suspension into a separate 1.5 mL eppendorf and add 290 μL Cell Isolation buffer. This aliquot will be used for the unstained and single stain samples.

***Note:*** This sample can be split further into more eppendorfs to use as single stain and unstained controls for flow cytometry and FACS-sorting.

21. Add 5 μL of the antibody mastermix to the remaining 100 μL cell suspension, mix gently, and incubate for 20 min at 4°C in the dark.

***Note:*** It is highly recommended to prepare flow cytometry single-stains by incubating leftover cells or samples with 1μl of each antibody (1:100 dilution). Also prepare an unstained sample to be used for gate determination during FACS sorting.

22. Wash the cell suspension with 1 mL Cell Isolation buffer.
23. Centrifuge at 300 **×** *g* for 5 min at 4°C and discard supernatant.
24. Resuspend the cell pellet with 300 μL Cell Isolation buffer and pass the sample through a 5 mL Falcon® Round-Bottom Tube with Cell Strainer Cap (35 μm nylon mesh) to ensure it is ready for FACS sorting. Keep samples at 4°C in the dark.
25. Samples are now ready for flow cytometry analysis and FACS sorting. FACS sorting should be performed within the next 4 h, but ideally as soon as possible.
26. Prepare 1.5 mL eppendorfs with 300 μL Sorting buffer (DMEM/10% FBS) and store on ice until use.

**Pause point:** The stained cells can be kept for 4 h at 4°C in the dark before FACS sorting. However, we suggest proceeding to FACS sorting as quickly as possible to maximize cell viability and recovery of fragile BM stromal cell subpopulations.

### FACS sorting of BM stromal cells and gating strategy

**Timing: ∼30 min**–**1 h, depending on number of cells in sample**

The purpose of FACS-sorting is to identify a variety of BM stromal cell subpopulations from large, heterogeneous BM samples. We characterize these BM stromal cells as CD45^-^ CD235a^-^ CD71^-^ CD41^-^ CD61^-^ cells on a flow cytometer in order to perform downstream analysis such as single-cell RNA sequencing on patient stromal cells. We stain for specific "contaminating” hematopoietic, megakaryocytic, and erythroid populations to be able to exclude these cells when sorting. Negative selection approaches are commonly used to enrich MSCs from mouse and patient samples (Baryawno et al., 2019), however, previous bulk RNA-sequencing strategies required additional stromal cell markers (Kenswil et al., 2021, Ping et al., 2019). The resolution of single cell analysis now allows for a more unbiased approach that does not require these markers. CD45 is used to identify the majority of hematopoietic cells. CD235a and CD71 (transferrin receptor) mark mature and progenitor erythroid cells, and CD41 and CD61 are used to identify mature and progenitor megakaryocytes.

**Figure 3:**
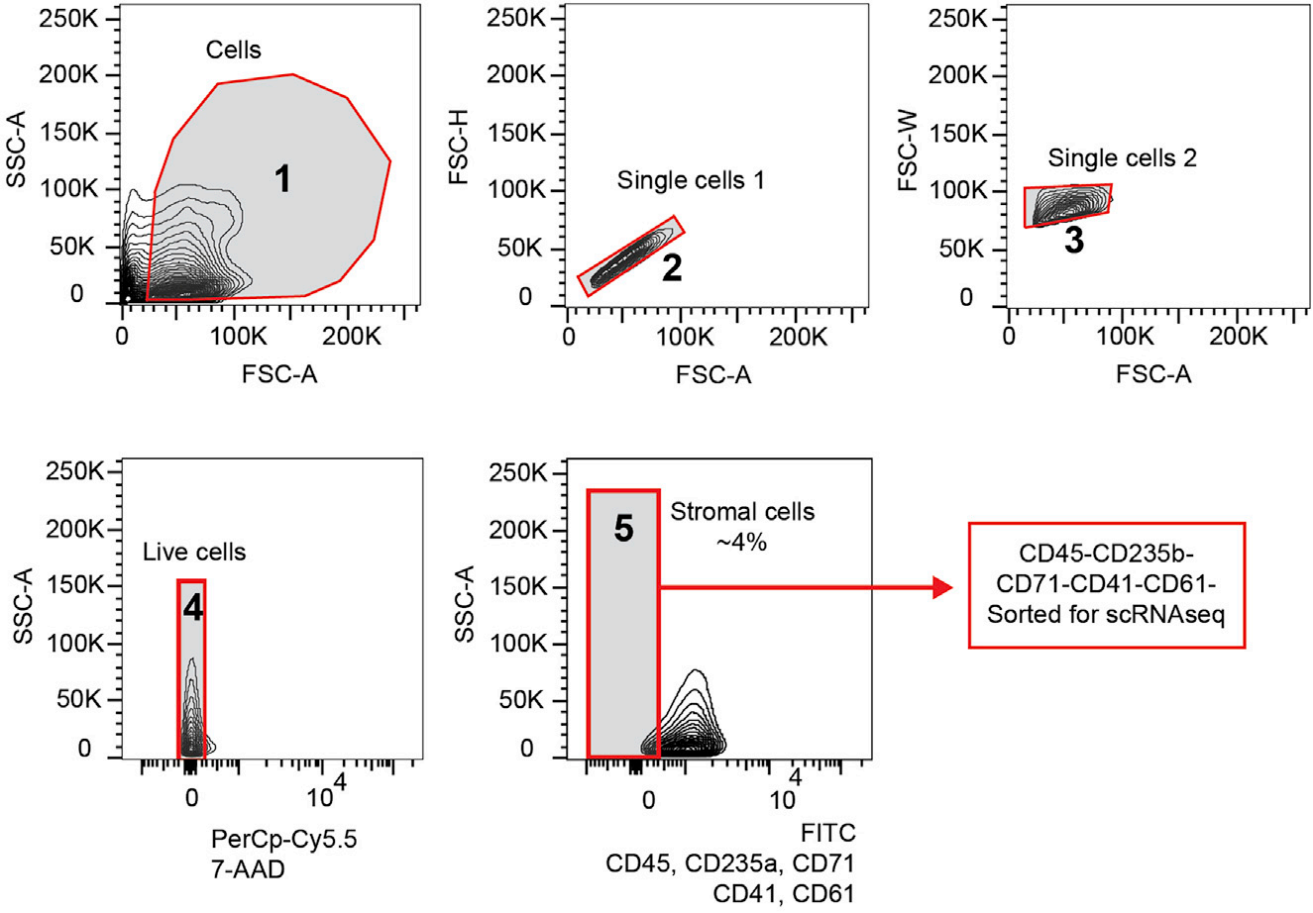
Sorting strategy for human BM stromal cell populations In panel 1, FSC versus SSC gating is used to identify cells of interest based on size and granularity, and then gated for single cells in panels 2 and 3. We subsequently select for live cells based on 7-AAD negativity (Panel 4, PerCp-Cy5.5 negative cells). Stromal cells are sorted by gating the negative populations (Panel 5) from the remaining cells that are stained for hematopoietic, megakaryocytic, and erythroid markers. FSC: forward scatter, used for the discrimination of cells by size, SSC: side scatter, provides information on the internal complexity, or granularity, of the cell. 7-AAD: 7-Aminoactinomycin D, FITC: Fluorescein isothiocyanate, scRNA-seq: single-cell RNA sequencing,

***Note:*** We use aliquots of single-stained cells for voltage settings, area scaling, and setting sort gates.

***Note:*** The 7-AAD solution is added just prior to FACS in order to prevent overstaining that can occur with prolonged exposure (no more than 1 h) of cells to 7-AAD solution.

27. Add 3 μL of 7-AAD solution (1:100 dilution) to the cell suspension just prior to cell sorting.
28. Gating strategy is provided in Figure 3.

29. Discard doublets by using FSC-H and FSC-W (Figure 3).
30. Discard non-viable cells using 7-AAD (PerCp-Cy5.5 channel).
31. Sort the desired CD45^-^ CD235a^-^ CD71^-^ CD41^-^ CD61^-^ cells (FITC channel) according to the gating strategy (Figure 3) into 300 μL sorting buffer (DMEM/2% FBS) in the prepared 1.5 mL eppendorfs and transfer onto ice.

***Note:*** From our experience on the BD FACS Aria II, we recommend using a 100μm nozzle for sorting. This places less pressure on sensitive cells like BM stromal cells and preserves viability. Additionally, we suggest sorting at a flow rate below 7,000 events/s, although flow rate will vary depending on the sorter type, sorter setup, and sorting efficiency.

32. Centrifuge collected cells at 300 **×** *g* for 5 min at 4°C.
33. In order to retain as many cells as possible for analysis, carefully remove the supernatant until you have approximately 40 μL left, then resuspend the cell pellet and proceed with the single-cell RNA sequencing pipeline.

***Optional:*** Perform cell count and viability check using a Countess II FL Automated Cell Counter (or alternative). However, it is likely that the number of sorted cells will be very small and hard to quantify. Performing a cell count at this stage also reduces the number of cells that can be analyzed for single-cell RNA sequencing, and we often omit this step and proceed directly to 10**×** Genomics, or alternative single-cell RNA sequencing pipeline.

**Pause point:** If more than one biopsy is isolated on the same day, sorted cells can be stored on ice until further processing. It is however, important to minimize sorting time and time between sorting and further processing as much as possible to keep sorted cells viable.

## Expected outcomes

Using this protocol, stromal cells are enriched from BM trephine biopsies (≥ 3 mm) after mechanical (flushing/crushing) and enzymatic treatment (using Collagenase II to disrupt collagen fibers), followed by flow-based cell sorting. Patient samples are highly variable but we have seen anywhere from 1%–4% stromal cells as shown in Figure 3. Specifically, the use of CD45^–^/CD71^–^/CD41^–^/CD61^–^/CD235a^–^/7AAD^–^ cells in single-cell RNA sequencing allows us to identify BM stromal cell subpopulations such as mesenchymal stromal cells (MSCs), fibroblasts, Schwann cells, small quantities of megakaryocyte precursors and a small population of myeloid/granulocytic cells (Leimkühler et al., 2020).

We have been able to consistently isolate over 13,000 droplets from 3 mm biopsies processed within 2 h of sample procurement using FACS-analysis, which translates to approximately 1,500 cells being sequenced appropriately for downstream RNA sequencing analysis. We hypothesize that newly single-cell RNA pipelines, and improved versions of existing single-cell RNA seq pipelines, such as 10**×** Genomics version 3 and higher, will recover even more cells than shown in this protocol.

## Limitations

One of the major challenges in late-stage myelofibrosis is that accessibility of liquid BM by aspiration is often limited (“dry tap”, or the inability to obtain liquid BM during marrow aspiration), and hematopoietic tissue for diagnosis is only available in the form of a BM biopsy. BM biopsies are highly variable between patients and might also vary depending on the location of the biopsy. Hence, it is difficult to obtain standardized results.

The combination of the mechanical and enzymatic release using Collagenase II of BM stromal cells is critical to the isolation of a variety of cell subpopulations detected downstream, but may also result in a higher density of “contaminating” hematopoietic cells. However, it is possible to computationally remove these “contaminating” populations from analysis after clustering and annotation during single-cell RNA sequencing data analysis.

The recovery of human MSC populations from patients with hematological malignancies for downstream analysis such as single-cell RNA sequencing is primarily hindered by low availability of patient material and the poor viability of BM stromal cells. We therefore established a protocol taking the experience of various mouse and human MSC isolation protocols into account (Baustian et al., 2015, Busser et al., 2015) as well as our own (Leimkühler et al., 2020, Schneider et al., 2017), to effectively extract MSCs from small BM biopsies. The development of this protocol was based on our experience with isolation of mouse MSCs, but optimized to yield the highest number of stromal cells and still maintain viability in a disease context, which massively alters BM structure.

The number of BM mesenchymal stromal cells recovered after this protocol is dependent on biopsy quality, biopsy size, and how long after biopsy the sample is processed. A biopsy that is too old or too small will result in smaller cell yields or in a significant reduction in cell viability. We have successfully isolated BM mesenchymal stromal cells from biopsies as small as 3 mm from excess material not needed for diagnostics. However, small numbers of cells can still be suitable for single-cell RNA sequencing and yield adequate numbers of cell populations for downstream analysis.

## Troubleshooting

### Problem 1

There are cell clumps when resuspending cell pellets (step 9).

### Potential solution

It is critical that single-cell suspensions be obtained before flow cytometry and FACS to prevent clogging on the cytometer. The most common reason for cell clumping is the presence of cell debris and free DNA in the solution, likely from excessive cell death. Handle cells with care and use the appropriate centrifuge settings.

Minor clumping can be resolved by trituration, or gentle up and down pipetting of cells.

Alternatively, the use of DNAse I (final concentration: 100 μg/mL, DN25-100 MG, Sigma-Aldrich) during biopsy disaggregation could also prevent clumping.

In addition, cell suspensions can also be passed through a new 70 μm cell strainer to remove cell debris that cannot be resuspended, although this will reduce the number of cells available for downstream analysis.

### Problem 2

Insufficient numbers or poor viability of stromal cells for downstream analysis (step 33).

### Potential solution

The number of BM mesenchymal stromal cells recovered after this protocol is dependent on biopsy quality, biopsy size, and how long after biopsy the sample is processed. Although a biopsy that is too old or too small will result in smaller cell yields, we have had success in isolating stromal cells from biopsies as small as 3 mm. In our hands, a 3 mm biopsy can yield 13,000 droplets from FACS-sorting and generate good quality single-cell RNA sequencing data for approximately 1,500 cells. Make sure that all cell solutions are kept at 4°C, except during collagenase II digestion, and process samples as quickly as possible.

### Problem 3

The sample has been stored for too long (>6 h) before transferred to lab and the biopsy is dried out (before you begin, step 1).

### Potential solution

Unfortunately, this is a risk with biopsies and leftover samples, which are not always transferred to the lab right away. We aim to start processing biopsies within 2 h of a sample being taken. A dried-out biopsy will severely reduce the number of cells that are recoverable and may not be suitable for single-cell RNA sequencing. We recommend discarding the dried-out biopsy and using a new sample from a different patient. Alternatively, it may be suitable to place the biopsy in PBS + 10% FBS and keep at 4°C until processing is possible, but we have not yet tested this out and have no data on the quality of the stromal cells extracted.

### Problem 4

Very few cells are detected on the FACS sorter or very few stromal cells are detected within the gate (steps 30 and 31).

### Potential solution

This problem may be due to the quality of the biopsy, but it is possible to sort all viable cells instead of removing “contaminating” hematopoietic cells (steps 30 and 31). In this manner, it is possible to capture the stromal cell populations of interest, as well as other cells, which can be removed from downstream analysis computationally.

### Problem 5

No positive staining of extracellular markers by flow cytometry (step 28).

### Potential solution

If no positive staining of extracellular markers is detected, this may be due to:

Antibody of interest was not added to the mastermix (before you begin, step 1). Ensure the antibody is added when repeating the experiment.

Antibody clone is not suitable. A different antibody clone can be used to select for certain populations, or additional markers can be used to enrich different stromal cell populations if desired.

We further recommend using single-stain and unstained controls to appropriately select the correct PMT voltages on the FACS sorter.

As also described in solution to problem 4, it is also possible to only sort viable cells, without additional markers, although this will result in a more heterogeneous population.

## Resource availability

### Lead contact

Further information and requests for resources and reagents should be directed to and will be fulfilled by the lead contact, Rebekka K. Schneider, MD, PhD (reschneider@ukaachen.de).

### Materials availability

This study did not generate new unique reagents. For specific details on availability please refer to the Key Resources Table.

### Data and code availability

This protocol did not generate/analyze datasets or code.
